# A nutraceutical product, extracted from *Cannabis sativa*, modulates voltage-gated sodium channel function

**DOI:** 10.1186/s42238-022-00136-x

**Published:** 2022-06-10

**Authors:** Carol J. Milligan, Lyndsey L. Anderson, Michael T. Bowen, Samuel D. Banister, Iain S. McGregor, Jonathon C. Arnold, Steven Petrou

**Affiliations:** 1 Florey Institute of Neuroscience and Mental Health, The University of Melbourne, Melbourne, VIC 3010 Australia; 2 Brain and Mind Centre, The University of Sydney, Sydney, Australia; 3 Lambert Initiative for Cannabinoid Therapeutics, The University of Sydney, Sydney, Australia; 4 Discipline of Pharmacology, Faculty of Medicine and Health, The University of Sydney, Sydney, Australia; 5 Department of Medicine, The University of Melbourne, Melbourne, VIC 3010 Australia

**Keywords:** Cannabidiol, Hemp seed oil, Phytocannabinoids, Terpenes, Entourage effect, Voltage-gated sodium channels

## Abstract

**Background:**

Purified cannabidiol (CBD), a non-psychoactive phytocannabinoid, has gained regulatory approval to treat intractable childhood epilepsies. Despite this, artisanal and commercial CBD-dominant hemp-based products continue to be used by epilepsy patients. Notably, the CBD doses used in these latter products are much lower than that found to be effective in reducing seizures in clinical trials with purified CBD. This might be because these CBD-dominant hemp products contain other bioactive compounds, including phytocannabinoids and terpenes, which may exert unique effects on epilepsy-relevant drug targets. Voltage-gated sodium (Na_V_) channels are vital for initiation of neuronal action potential propagation and genetic mutations in these channels result in epilepsy phenotypes. Recent studies suggest that Na_V_ channels are inhibited by purified CBD. However, the effect of cannabis-based products on the function of Na_V_ channels is unknown.

**Methods:**

Using automated-planar patch-clamp technology, we profile a hemp-derived nutraceutical product (NP) against human Na_V_1.1–Na_V_1.8 expressed in mammalian cells to examine effects on the biophysical properties of channel conductance, steady-state fast inactivation and recovery from fast inactivation.

**Results:**

NP modifies peak current amplitude of the Na_V_1.1–Na_V_1.7 subtypes and has variable effects on the biophysical properties for all channel subtypes tested. NP potently inhibits Na_V_ channels revealing half-maximal inhibitory concentration (IC_50_) values of between 1.6 and 4.2 μg NP/mL. Purified CBD inhibits Na_V_1.1, Na_V_1.2, Na_V_1.6 and Na_V_1.7 to reveal IC_50_ values in the micromolar range. The CBD content of the product equates to IC_50_ values (93–245 nM), which are at least an order of magnitude lower than purified CBD. Unlike NP, hemp seed oil vehicle alone did not inhibit Na_V_ channels, suggesting that the inhibitory effects of NP are independent of hemp seed oil.

**Conclusions:**

This CBD-dominant NP potently inhibits Na_V_ channels. Future study of the individual elements of NP, including phytocannabinoids and terpenes, may reveal a potent individual component or that its components interact to modulate Na_V_ channels.

**Supplementary Information:**

The online version contains supplementary material available at 10.1186/s42238-022-00136-x.

## Background

Nearly two-thirds of epilepsies are classified as being genetic in origin (Berkovic et al. 2006; Miller et al. 1998; Speed et al. 2014). Advances in genome sequencing have enabled the systematic screening of patient genomes to identify mutations in single genes that are responsible for epilepsy (Perucca and Perucca 2019). These monogenic epilepsies all report mutations in molecular components of neuronal signalling, with mutations in Na_V_ channels being the most prevalent (Parrini et al. 2017). Functional characterization of the biophysical properties of these disease-associated mutant channels has provided insight on the molecular mechanisms by which Na_V_ channel dysfunction contributes to epileptogenesis. Na_V_ channel mutations that have been identified exhibit altered biophysical properties representative of both gain-of-function (GOF) and loss-of-function (LOF) phenotypes (Berecki et al. 2019; Berecki et al. 2018; Blanchard et al. 2015; de Kovel et al. 2014).

Mutations in *SCN1A*, the gene that encodes voltage-gated sodium channel Na_V_1.1, account for the greatest number of mutations identified and range in severity across various epilepsy types (Depienne et al. 2009; Djemie et al. 2016). De novo LOF mutations in *SCN1A* are present in over 80% of patients with Dravet syndrome (DS), a severe form of childhood-onset epilepsy. As Na_V_1.1 channels are expressed predominantly in GABAergic interneurons, LOF mutations result in reduced interneuron activity, thereby causing an imbalance between excitatory and inhibitory neurotransmission (Brunklaus and Zuberi 2014). Mutations in *SCN2A* and *SCN8A*, the genes that encode Na_V_1.2 and Na_V_1.6, respectively, have been implicated in Lennox-Gastaut syndrome (LGS), another severe childhood epilepsy (Epi4K. 2013). Na_V_1.2 and Na_V_1.6 are the primary Na_V_ channels expressed in excitatory pyramidal neurons (Hu et al. 2009). Functional studies indicate that the majority of *SCN2A* and *SCN8A* mutations are GOF, resulting in neuronal hyperexcitability (Epi4K. 2013; Estacion et al. 2014; Veeramah et al. 2012).

Many epilepsy syndromes, including DS and LGS, are resistant to current treatments creating an urgent need for novel therapies with improved side effect profiles. Cannabis-based products are increasingly being used in intractable epilepsies (Suraev et al. 2018; Suraev et al. 2017), following numerous media stories featuring significant improvements in treatment-resistant childhood epilepsy patients using medicinal cannabis. These artisanal and commercially manufactured products contain hundreds of potentially bioactive compounds, including phytocannabinoids and terpenoids. Both phytocannabinoids and terpenes modulate a range of membrane proteins, including ion channels, and have exhibited anticonvulsant properties in preclinical seizure models (Anderson et al. 2019a, b; Gray and Whalley 2020). A purified preparation of the non-psychoactive phytocannabinoid CBD, Epidiolex™, was approved by drug regulatory agencies in the USA, Europe and Australia for the treatment of DS, LGS and tuberous sclerosis complex. While the anticonvulsant mode of action of CBD is unclear, cellular studies have shown that CBD affects several ion channels, such as 5-HT_1A_R, TRP channels, T-type calcium channels, GABA_A_ receptors and Na_V_ channels (Ghovanloo et al. 2018; Watkins 2019).

Recent studies suggest that Na_V_ channel currents (Na_V_1.1-Na_V_1.7) are attenuated by purified CBD (Duan et al. 2008; Ghovanloo et al. 2018; Okada et al. 2005). However, the effect of cannabis-based products on the function of Na_V_ channels is unknown. Here we use automated-planar patch-clamp technology to characterize the effects of a nutraceutical product (NP) against the human voltage-gated sodium channel family (Na_V_1.1-Na_V_1.8). We examined the effects of NP on the biophysical properties of channel conductance, steady-state fast inactivation and recovery from fast inactivation. Additionally, on the same biophysical parameters, we characterized the effects of a hemp seed oil preparation that contained the natural terpenes but was devoid of phytocannabinoids.

## Methods

### Tissue culture and transfection

HEK293T cells stably expressing *SCN1A*, *SCN2A*, *SCN3A*, *SCN5A* or *SCN9A* and CHO cells stably expressing *SCN4A* or *SCN8A* were maintained as previously described (Richards et al. 2018). CHO cells were transiently co-transfected with the pcDNA3.1-*SCN10A* construct as previously described (Knapp et al. 2012) and a GFP construct, using Lipofectamine 3000, according to the manufacturer’s instructions (Thermofisher).

### Planar patch-clamp electrophysiology

Patch-clamp recordings were made using a Patchliner® (Nanion Technologies, Munich, Germany) in the whole-cell configuration as previously described (Richards et al. 2018). Briefly, cells were prepared in suspension at a density of 1×10^6^–5x10^7^ cells/mL. The external recording solution comprised (in mM): 140 NaCl, 4 KCl, 1 MgCl_2_, 2 CaCl_2_, 5 D-glucose, 10 HEPES, pH 7.4 with NaOH, ~295 mOsm. The internal recording solution comprised (in mM): 50 CsCl, 60 CsF, 10 NaCl, 20 EGTA, 10 HEPES, pH 7.2 with CsOH, ~285 mOsm. NP was diluted in DMSO. Medium single-hole planar -16 chips with an average resistance of ~2.5 MΩ were used. Chip and whole-cell capacitance were fully compensated, and 50% series resistance compensation applied. Recordings were acquired at 50 kHz with the low pass filter set to 10 kHz in PATCHMASTER (HEKA Instruments, NY, USA) and performed at 27°C. Offline analysis was performed using Microsoft Excel, MatLab R2019a (MathWorks) and GraphPad Prism 8 (Molecular Devices). Biophysical analysis was performed using MatLab scripts, incorporating the curve fitting equations described below, tailored to enable analysis of multiple-cell data output acquired using automated-planar patch-clamp technology.

### Voltage clamp protocols

Voltage protocols were used, as previously described (Richards et al. 2018). Briefly, to study the voltage-dependence of activation, cells were held at −120 mV and depolarized to test potentials, in 5 mV increments, between −120 and +50 mV for 100 ms. To study steady-state fast inactivation, cells were held at conditioning pre-pulse potentials ranging from −120 to +30 mV in 5 mV increments from a holding potential of −120 mV and a test pulse at 5 mV for 20 ms. Recovery from fast inactivation was studied by pre-pulsing the cells to 0 mV from a holding potential of −120 mV for 50 ms, to fully inactivate channels. The voltage was then stepped back to the holding potential for variable interpulse intervals (ipi from 0 to 39 ms in 3 ms increments). To test channel availability, the voltage was stepped to 0 mV for 50 ms. To determine IC_50_ values for NP, hemp seed oil and purified CBD, cells were held at −80 mV, stepped to −120 mV for 200 ms followed by 50 ms test depolarization to 0 mV every 2 s for 30 s in the presence of vehicle control (DMSO). The cells were then exposed to NP (0.03–9 μg NP/mL), hemp seed oil (0.03–9 μg hemp seed oil/mL) or purified CBD (0.1–100 μM) sequentially for 5 min. Currents for individual cells were averaged over 24-s periods directly before application and following a 5-min exposure of NP, hemp seed oil or purified CBD. Leak subtraction was applied before normalization of current amplitude. Normalized mean data were fit to the Hill equation.

### Curve fitting and data analysis

To examine the voltage-dependence of activation, normalized current-voltage (*I-V*) relationships were converted to conductance (*G*) using the following equation: *G*=*I*/(*V-V*_r_) where *V*_r_ is the reversal potential for Na^+^. The voltage-dependence of conductance and availability were normalized and fitted to a Boltzmann equation: *G*=1/(1+exp[(*V*-*V*_0.5_)/*a*]), where *a* is the slope of the half-maximum, *V* is the potential of the given pulse and *V*_0.5_ is the potential for the half-maximal activation/inactivation. To measure recovery from inactivation, normalized currents were plotted against ipi and data fitted with the equation *I/I*_max_=1−exp/(*rc*+*x*), where *I*_max_ is maximal current; *rc* recovery rate constant; *x* is time. The time course of inactivation was fitted to a single exponential function *I/Imax*=*I*_0_+A*exp(-*t*-*t*0/*τ*)+*C*, where *I*_0_ is the non-inactivating component, *Imax* is the peak current, *t* is time and *A* is the component for the time constant *τ*. Time constants were plotted against voltage and the data fitted with a decaying exponential equation *Y*=span*exp(-K*x)+*plateau*, where *span* is the starting point of the curve, *K* is the decay factor, *plateau* is the value the curve decays to and *x* is time.

### Nutraceutical product

The nutraceutical product (NP), Ananda Hemp 600 and hemp seed oil were provided for free by Ananda Professional Pty Ltd. NP was produced by ethanol extraction of hemp and stored at 4°C. Experiments were performed over a 4-month period, which complies with the 12-month shelf life, and comparable bioactive effects were observed throughout the series of experiments. The cold-pressed hemp seed oil was devoid of phytocannabinoids and terpenoids. The NP contained the following phytocannabinoids (mg/g product): cannabidiolic acid (CBDA) 1.4, cannabidiol (CBD) 18.3, cannabinol (CBN) 0.3 and Δ^9^-tetrahydrocannabinol (Δ^9^-THC) 3 (Additional file 1). Further characterization by LC-MS/MS (Suraev et al. 2018) showed cannabidivarinic acid (CBDVA), cannabidivarin (CBDV), cannabigerolic acid (CBGA), cannabigerol (CBG), Δ^9^-tetrahydrocannabinolic acid (THCA), Δ^9^-tetrahydrocannabidvarinic acid (THCVA), Δ^9^-tetrahydrocannabidvarin (THCV) and cannabichromene (CBC) were not present. Analysis by GC-MS (Suraev et al. 2018) showed the NP had the following terpene content (mg/g hemp seed oil): α-pinene 1.6, D-limonene 0.6, β-linalool 2.1 and β-caryophyllene 3.1. The highest concentration tested (9 μg NP/mL), equates to molar exposures of (nM): CBDA 35, CBD 520, CBN 9, Δ^9^-THC 86, α-pinene 106, D-limonene 40, β-linalool 60 and β-caryophyllene 137. Final drug concentrations contained 0.1% DMSO.

### Statistical analyses

All statistical analyses were performed using GraphPad Prism 8 (Molecular Devices) software, with a *p* value <0.05 considered statistically significant. Data values are expressed as mean±SEM. Comparisons were made between the vehicle and extract datasets using paired Student’s *t* tests. Comparisons were also made between IC_50_ values for pure CBD and the equivalent molar IC_50_ of CBD found in NP using unpaired Student’s *t* tests.

## Results

Previous studies show that CBD is able to modulate the function of human Na_V_ channels; however, data showing how commercially available phytocannabinoid preparations affect these ion channels is lacking (Duan et al. 2008; Ghovanloo et al. 2018; Okada et al. 2005). Here, we examined the impact of an orally administered phytocannabinoid-containing nutraceutical product (NP), on eight human voltage-dependent sodium channel subtypes, Na_V_1.1-Na_V_1.8. We examined the impact of NP on the biophysical properties of each channel and determined potency.

### NP inhibits peak sodium currents

**Fig. 1:**
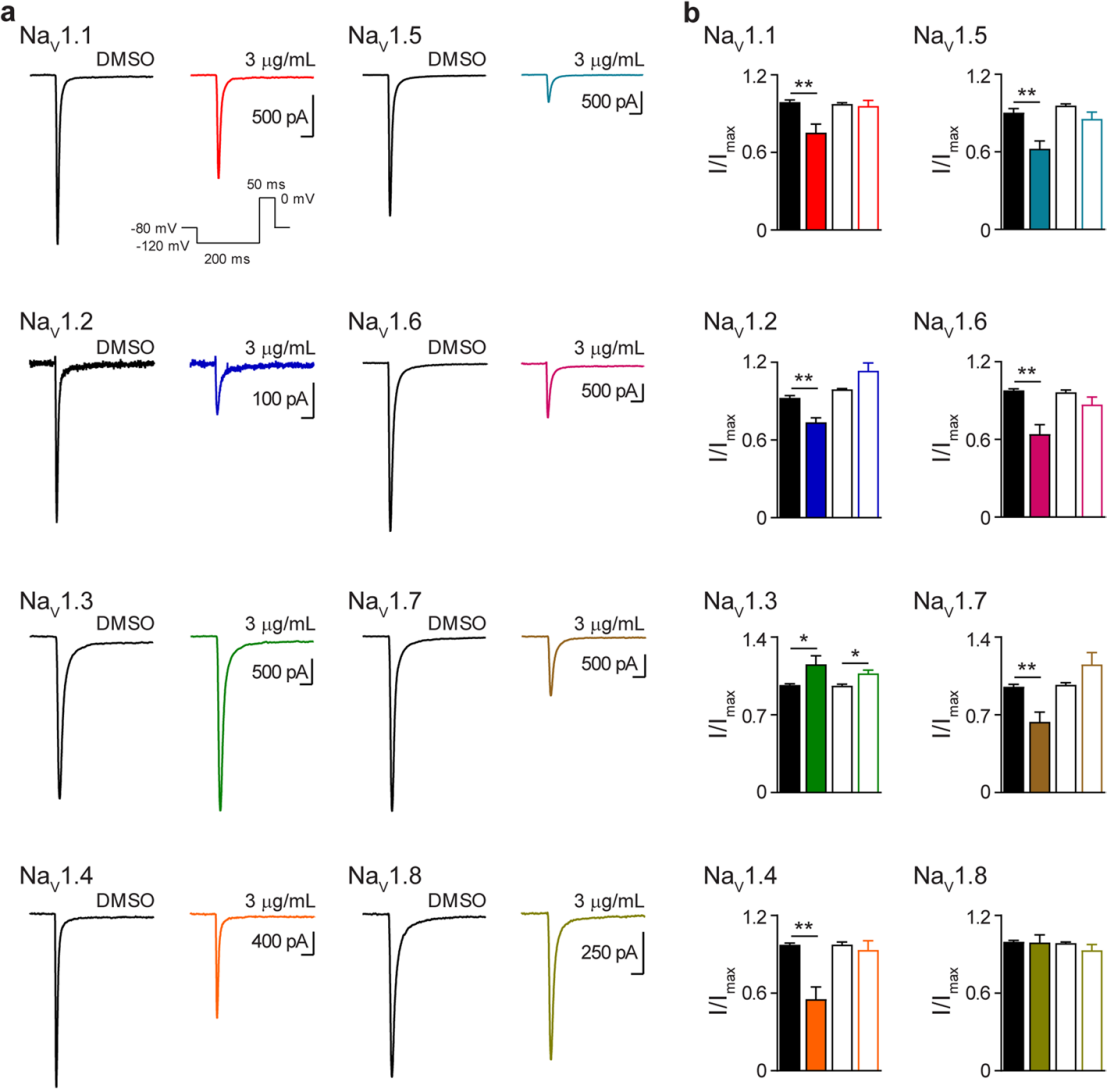
NP inhibits peak current amplitude of Na_V_ channels. **a** Representative current traces, evoked by the indicated voltage protocol (inset), in the presence of DMSO vehicle (  ) or NP (3 μg NP/mL) for Na_V_1.1 (  ), Na_V_1.2 (  ), Na_V_1.3 (  ), Na_V_1.4 (  ), Na_V_1.5 (  ), Na_V_1.6 (  ), Na_V_1.7 (  ) and Na_V_1.8 (  ) expressed in HEK 293 or CHO cells. Horizontal scale bars (2 ms) apply to all traces. **b** Mean normalized peak current amplitude (I/I_max_) in the presence of DMSO vehicle (  ) or NP (3 μg NP/mL) for Na_V_1.1 (  ; *n*=10), Na_V_1.2 (  ; *n*=8), Na_V_1.3 (  ; *n*=10), Na_V_1.4 (  ; *n*=6), Na_V_1.5 (  ; *n*=10), Na_V_1.6 (  ; *n*=11), Na_V_1.7 (  ; *n*=7), Na_V_1.8 (  ; *n*=7) and DMSO vehicle (  or, hemp seed oil (3 μg hemp seed oil/mL) for Na_V_1.1 (  ; *n*=9), Na_V_1.2 (  ; *n*=5), Na_V_1.3 (  ; *n*=5), Na_V_1.4 (  ; *n*=9), Na_V_1.5 (  ; *n*=5), Na_V_1.6 (  ; *n*=5), Na_V_1.7 (  ; *n*=11) and Na_V_1.8 (  ; *n*=7). Data points are mean±SEM. Peak current amplitudes in the presence of NP were compared to DMSO vehicle (  ) currents. Peak current amplitudes in the presence of hemp seed oil were compared to DMSO vehicle (  ) currents. Statistical significance is marked as *p<0.05; **p<0.01

Whole-cell current recordings were recorded from cells expressing a single Na_V_ isoform using automated-planar patch-clamp technology. NP (3 μg NP/mL) significantly inhibited the peak amplitude of sodium currents elicited by six of the Na_V_ channel subtypes Na_V_1.1 (peak current (nA): DMSO −1.8±0.4, NP −1.3±0.3, *p*=0.01), Na_V_1.2 (peak current (nA): DMSO −1.0±0.2, NP −0.8±0.2, *p*=0.006), Na_V_1.4 (peak current (nA): DMSO −4.4±1.1, NP −3.0±1.1, *p*=0.002), Na_V_1.5 (peak current (nA): DMSO −8.2±1.0, NP −5.9±1.0, *p*=0.0004), Na_V_1.6 (peak current (nA): DMSO −1.5±0.3, NP −1.0±0.3, *p*=0.03) and Na_V_1.7 (peak current (nA): DMSO −1.9±0.3, NP −1.2±0.2, *p*=0.03) (Fig. 1a, b). Na_V_1.8 currents were not affected by NP (Fig. 1a, b). Interestingly, at the same concentration, NP significantly potentiated Na_V_1.3 peak current amplitude (peak current (nA): DMSO −1.2±0.2, NP −1.4±0.2, *p*=0.04) (Fig. 1a, b). Sodium channel currents were recorded in the presence of 3 μg hemp seed oil/mL. Hemp seed oil had no effect on the peak current amplitude elicited by Na_V_1.1, Na_V_1.2, Na_V_1.4, Na_V_1.5, Na_V_1.6, Na_V_1.7 or Na_V_1.8 (Fig. 1b and Additional file 2). However, similar to what was seen with NP, hemp seed oil potentiated Na_V_1.3 peak current amplitude (peak current (nA): DMSO −0.9±0.2, Hemp −1.2±0.2, *p*=0.04) (Fig. 1b and Additional file 2).

### Divergent effects of NP on biophysical properties of Na_V_ channels

**Fig. 2:**
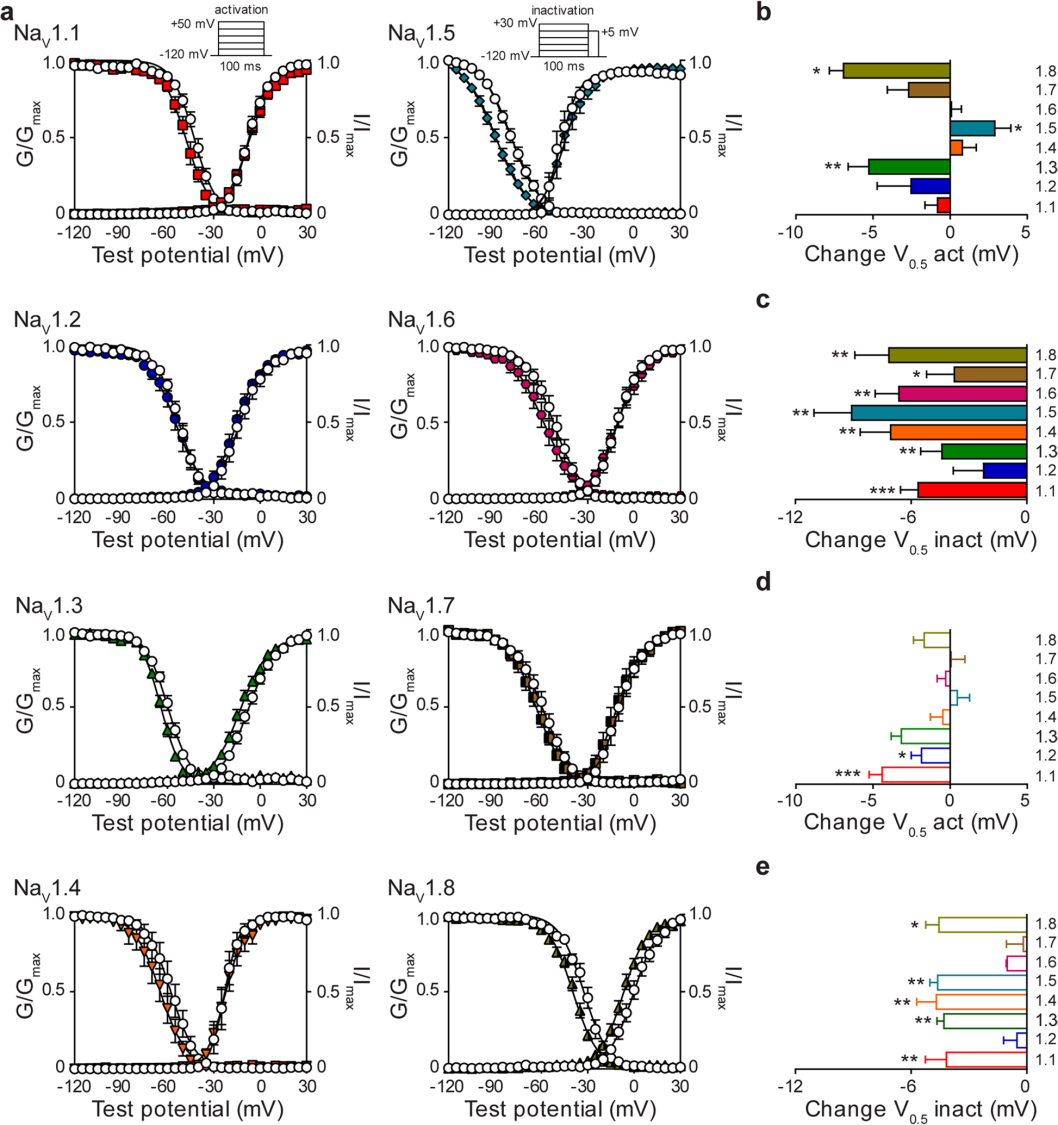
The effects of NP on activation and steady-state fast inactivation (SSFI). **a** Voltage-dependence of normalized peak conductance (G/G_max_) and SSFI (I/I_max_), evoked by the indicated voltage protocols (insets), in the presence of vehicle (  ) or NP (3 μg NP/mL) for Na_V_1.1 (  ; *n*=10; mean diff slope act 1.5±0.3, *p*=0.002), Na_V_1.2 (  ; *n*=8), Na_V_1.3 (  ; *n*=10), Na_V_1.4 (  ; *n*=6; mean diff slope act 1.6±0.6, *p*=0.04; mean diff slope inact 0.8±0.3, *p*=0.03), Na_V_1.5 (  ; *n*=10; mean diff slope act 2.1±0.5, *p*=0.003; mean diff slope inact 0.8±0.3, *p*=0.003), Na_V_1.6 (  ; *n*=11; mean diff slope act 1.3±0.4, *p*=0.01), Na_V_1.7 (  ; *n*=7) and Na_V_1.8 (  ; *n*=7). Boltzmann curves were fitted to pooled averages of peak conductance. **b** Average change in the voltage of half (V_0.5_) activation and **c** SSFI initiated by NP (3 μg NP/mL) for Na_V_1.1 (  ; *n*=10), Na_V_1.2 (  ; *n*=8), Na_V_1.3 (  ; *n*=10), Na_V_1.4 (  ; *n*=6), Na_V_1.5 (  ; *n*=10), Na_V_1.6 (  ; *n*=11), Na_V_1.7 (  ; *n*=7) and Na_V_1.8 (  ; *n*=7). **d** Average change in the voltage of half V_0.5_ activation and **e** SSFI caused by hemp seed oil (3 μg hemp seed oil/mL) for Na_V_1.1 (  ; *n*=11), Na_V_1.2 (  ; *n*=5), Na_V_1.3 (  ; *n*=5), Na_V_1.4 (  ; *n*=9), Na_V_1.5 (  ; *n*=6), Na_V_1.6 (  ; *n*=6), Na_V_1.7 (  ; *n*=10) and Na_V_1.8 (  ; *n*=5). Data points are mean±SEM. Statistical significance is marked as *p<0.05; **p<0.01; ***p<0.001

Next, we sought to determine whether NP affects the biophysical properties of channel activation and steady-state fast inactivation. Conductance curves were fitted with a Boltzmann equation, and the voltage-dependence of half-activation (V_0.5_) and slope values were obtained. No changes in the voltage-dependence of activation were observed for Na_V_1.1, Na_V_1.2, Na_V_1.4, Na_V_1.6 or Na_V_1.7 channel subtypes (Fig. 2a, b). Although there is no effect on the voltage-dependence of activation for Na_V_1.1, a small, but significant change in the slope of the conductance curve was observed (slope factor: DMSO 6.1±0.4, NP 7.6±0.6 *p*=0.002). This suggests that NP has a GOF effect on Na_V_1.1 since a larger slope factor indicates greater activation of the channel at voltages negative to V_0.5_. NP (3 μg NP/mL) treatment resulted in significant hyperpolarizing shifts in the voltage-dependence of activation for Na_V_1.3 (V_0.5_ act (mV): DMSO −7.3±1.7, NP −12.6±1.5, *p*=0.003) and Na_V_1.8 (V_0.5_ act (mV): DMSO −0.1±2.1, NP −7.0±1.3, *p*=0.02), consistent with an increase in channel availability (Fig. 2a, b). In contrast, a significant depolarizing shift in the voltage-dependence of activation was observed for Na_V_1.5 (V_0.5_ act (mV): DMSO −46.8±2.4, NP −43.8±2.6, *p*=0.02) following treatment with NP, which is consistent with a decrease in channel availability (Fig. 2a, b). Again, we examined the effect of hemp seed oil alone on voltage-dependence of activation for each Na_V_ isoform. Hemp seed oil caused small but significant hyperpolarizing shifts in the voltage-dependence of activation for Na_V_1.1 (V_0.5_ act (mV): DMSO −11.0±1.7, Hemp −15.4±1.5, *p*=0.002) and Na_V_1.2 (V_0.5_ act (mV): DMSO −12.6±2.1, Hemp −14.5±2.6, *p*=0.04) (Fig. 2d and Additional file 3). However, hemp seed oil did not affect the voltage-dependence of activation of the other sodium channel subtypes (Fig. 2d and Additional file 3). All Na_V_ isoforms, except Na_V_1.2, exhibited a negative shift in the voltage-dependence of steady-state fast inactivation (V_0.5_ inact (mV): Na_V_1.1 DMSO −42.6±1.5, NP −48.3±1.8, *p*=0.0001; Na_V_1.3 DMSO −59.7±1.2, NP −64.1±0.9, *p*=0.002; Na_V_1.4 DMSO −58.0±3.5, NP −65.1±3.8, *p*=0.006; Na_V_1.5 DMSO −79.3±1.7, NP −88.4±1.8, *p*=0.001; Na_V_1.6 DMSO −52.2±2.4, NP −58.7±3.1, *p*=0.0004; Na_V_1.7 DMSO −59.7±2.3, NP −63.5±2.1, *p*=0.03; Na_V_1.8 DMSO −32.4±1.8, NP −39.6±1.3, *p*=0.006) in the presence of NP (Fig. 2a, c). A hyperpolarizing shift in fast inactivation is indicative of reduced channel availability. Hemp seed oil also resulted in a negative shift in the voltage-dependence of fast inactivation for all isoforms (V_0.5_ inact (mV): Na_V_1.1 DMSO −48.7±1.4, Hemp −52.9±1.2, *p*=0.003; Na_V_1.3 DMSO −55.8±1.3, Hemp −60.1±1.3, *p*=0.002; Na_V_1.4 DMSO −56.7±0.7, Hemp −64.4±1.4, *p*=0.005; Na_V_1.5 DMSO −77.9±2.4, Hemp −80.6±2.4, *p*=0.003; Na_V_1.8 DMSO −41.1±1.8, Hemp −45.7±2.4, *p*=0.03) except Na_V_1.2, Na_V_1.6 and Na_V_1.7 (Fig. 2e and Additional file 3). Despite the effects on the voltage-dependence of inactivation, neither NP nor hemp seed oil affected the time constants of fast inactivation (Additional files 4 and 5).

**Fig. 3:**
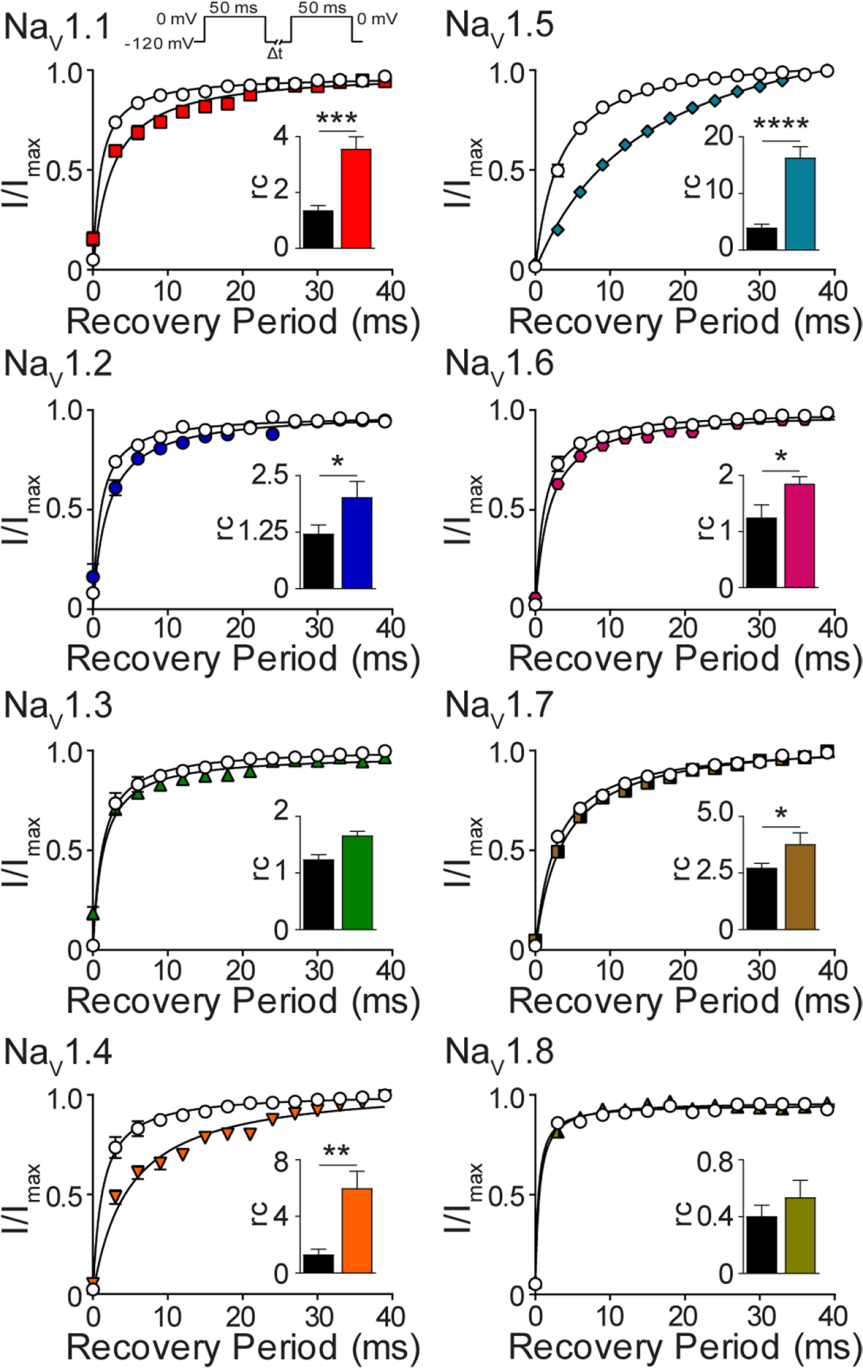
The effect of NP on sodium channel recovery from fast inactivation. Recovery of channel availability from fast inactivation as a function of time, evoked by the indicated voltage protocol (inset), in the presence of DMSO vehicle (  ) or NP (3 μg NP/mL) for Na_V_1.1 (  ; *n*=10), Na_V_1.2 (  ; *n*=8), Na_V_1.3 (  ; *n*=10), Na_V_1.4 (  ; *n*=6), Na_V_1.5 (  ; *n*=10), Na_V_1.6 (  ; *n*=11), Na_V_1.7 (  ; *n*=7) and Na_V_1.8 (  ; *n*=7). A hyperbola was fitted to pooled averages. Insets show the mean rc values in the presence of DMSO vehicle (black bars) or NP (coloured bars as described above). Data points are mean±SEM. Statistical significance is marked as *p <0.05, **p <0.01, ***p <0.001, ****p <0.0001

However, recovery from steady-state fast inactivation was significantly slower in the presence of NP for Na_V_1.1 (rc: DMSO 1.0±0.1, NP 2.7±0.3, *p*=0.0002), Na_V_1.2 (rc: DMSO 1.0±0.2, NP 2.0±0.3, *p*=0.01), Na_V_1.4 (rc: DMSO 1.4±0.4, NP 5.2±1.1, *p*=0.01), Na_V_1.5 (rc: DMSO 3.4±0.3, NP 16.3±1.9, *p*=0.0001), Na_V_1.6 (rc: DMSO 1.1±0.2, NP 1.6±0.1, *p*=0.01) and Na_V_1.7 (rc: DMSO 2.7±0.2, NP 3.8±0.5, *p*=0.03) (Fig. 3). A slower recovery from steady-state fast inactivation suggests reduced channel availability and is consistent with a decrease in channel activity. NP did not affect the recovery from steady-state fast inactivation for either Na_V_1.3 or Na_V_1.8 (Fig. 3). Hemp seed oil only slowed the recovery from fast inactivation of Na_V_1.5 (rc: DMSO 4.5±0.6, Hemp 7.0±1.2, *p*=0.005) and had no effect on the other channels (Additional file 6).

### NP potently inhibits voltage-gated sodium channels

**Fig. 4:**
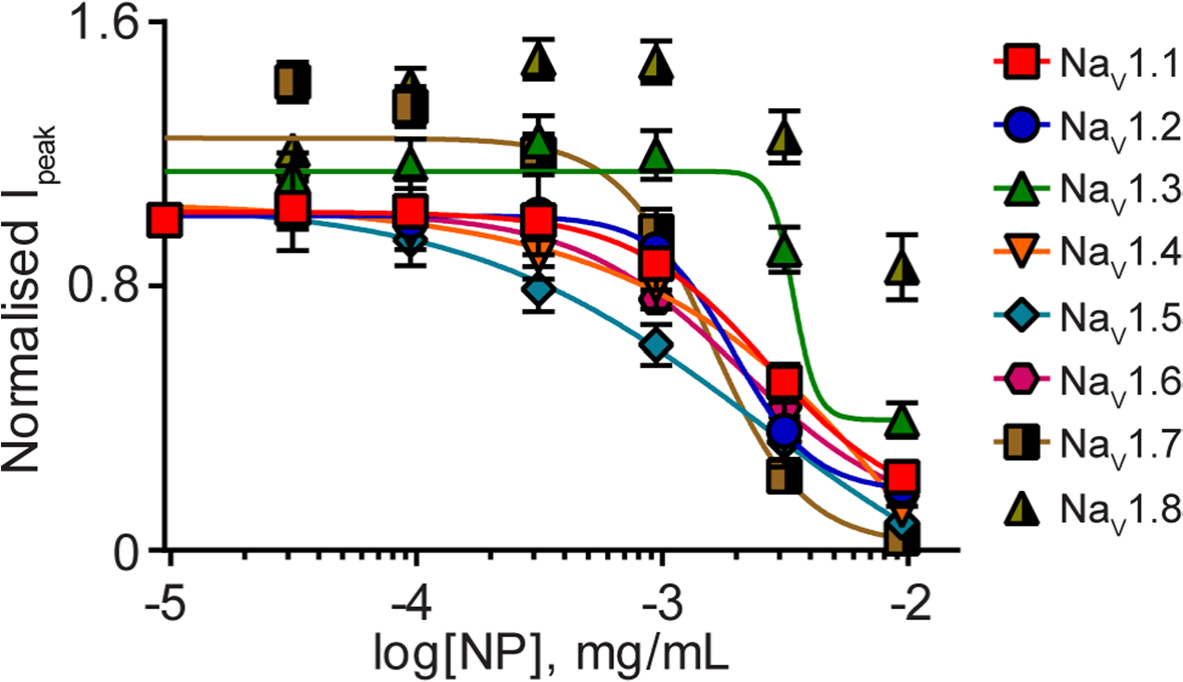
Variable potency of NP for the Na_V_1.1-Na_V_1.8 channels. Concentration-response curves for NP (0.03–9 μg NP/mL) against Na_V_1.1 (  ; *n*=6), Na_V_1.2 (  ; *n*=6), Na_V_1.3 (  ; *n*=6), Na_V_1.4 (  ; *n*=6), Na_V_1.5 (  ; *n*=6), Na_V_1.6 (  ; *n*=7), Na_V_1.7 (  ; *n*=8) and Na_V_1.8 (  ; *n*=6). Data points are mean±SEM

**Fig. 5:**
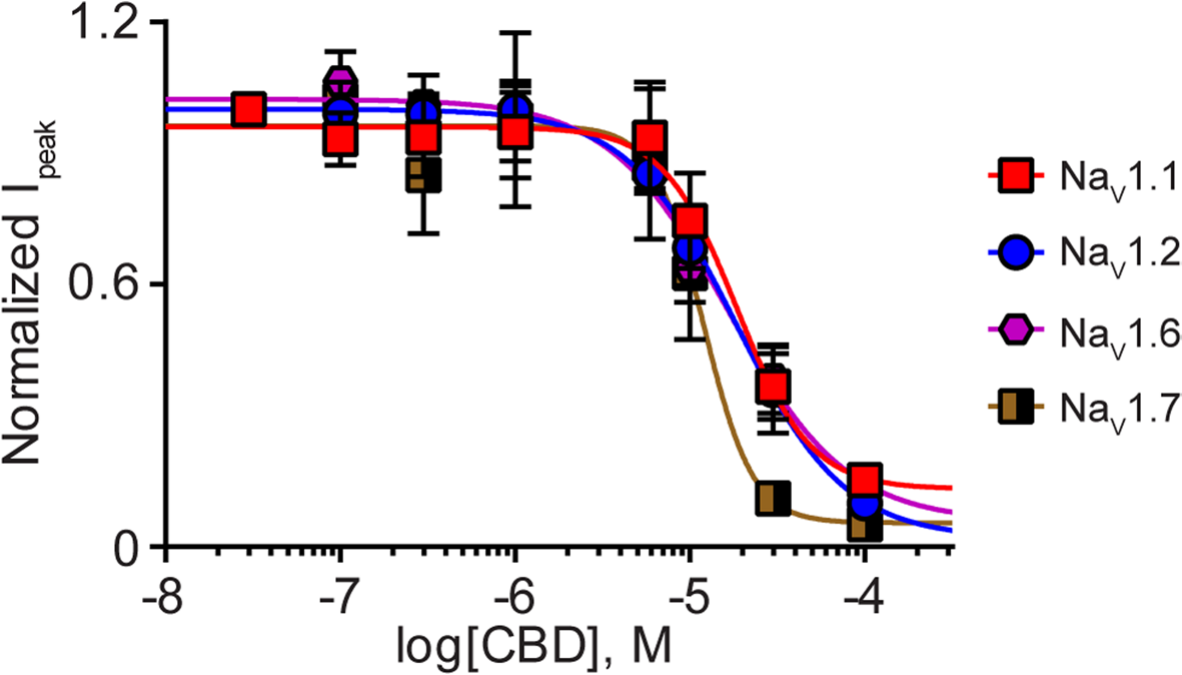
Variable potency of purified CBD for a subset of the Na_V_ channels. Concentration-response curves for purified CBD (0.1–100 μM) against Na_V_1.1 (  ; *n*=9), Na_V_1.2 (  ; *n*=6), Na_V_1.6 (  ; *n*=7) and Na_V_1.7 (  ; *n*=7). Data points are mean±SEM

Lastly, we determined the potency of NP on inhibition of peak current amplitude across all eight Na_V_ channel isoforms. We constructed concentration-response curves in cells sequentially exposed to NP (0.03–9 μg NP/mL) to calculate IC_50_ values (Fig. 4). NP potently inhibits sodium currents through all sodium channel subtypes, except Na_V_1.8, with IC_50_ values ranging from 1.6 to 4.2 μg NP/mL (Table 1). No concentration-dependent inhibition of peak current amplitude was observed with hemp seed oil for any of the channel isoforms (Additional file 7). As CBD was the most abundant cannabinoid in the NP, we determined the IC_50_ values of purified CBD at a subset of the sodium channels Na_V_1.1, Na_V_1.2, Na_V_1.6 and Na_V_1.7 (Fig. 5). The results revealed IC_50_ values ranging from 11.9 to 18.5 μM (Table 2). Using the IC_50_ values for NP, we derived the equivalent molar IC_50_ of CBD found in NP for individual cells and compared them with purified CBD IC_50_ values for the sodium channels Na_V_1.1 (IC_50_ (μM): NP 0.169±0.02, CBD 18.5± 2.2, *p*=0.0002), Na_V_1.2 (IC_50_ (μM): NP 0.245±0.03, CBD 18.4±2.6, *p*=0.0001), Na_V_1.6 (IC_50_ (μM): NP 0.099±0.02, CBD 16.6±1.8, *p*=0.001) and Na_V_1.7 (IC_50_ (μM): NP 0.095±0.001, CBD 11.9±2.2, *p*=0.0001).

**Table 1:** IC_50_ values for NP on Na_V_1.1-1.7 peak Na_V_ current and equivalent molar IC_50_ of CBD found in NP

Isoform	IC_**50**_ value (μg NP/mL)	CBD (nM)	n (cells)
Na_V_1.1	2.9 ± 0.7	169	6
Na_V_1.2	4.2 ± 0.3	245	6
Na_V_1.3	4.0 ± 0.2	233	6
Na_V_1.4	2.2 ± 0.3	128	6
Na_V_1.5	2.5 ± 0.3	146	6
Na_V_1.6	1.7 ± 0.3	99	7
Na_V_1.7	1.6 ± 0.1	93	8
Na_V_1.8	Not determined	-	6

IC_50_ values for NP are mean±SEM. *IC*_*50*_ half-maximal inhibitory concentration, *NP* nutraceutical product, *CBD* cannabidiol, *Na*_*V*_ Voltage-gated sodium

**Table 2:** IC_50_ values for CBD on a subset of the Na_V_ channel isoforms

Isoform	IC_**50**_ value (μM)	n (cells)
Na_V_1.1	18.5 ± 2.2	9
Na_V_1.2	18.4 ± 2.6	6
Na_V_1.6	16.6 ± 1.8	7
Na_V_1.7	11.9 ± 2.2	7

IC_50_ values are mean±SEM. *IC*_*50*_ half-maximal inhibitory concentration, *CBD* cannabidiol, *Na*_*V*_ voltage-gated sodium

## Discussion

Preclinical and clinical studies show CBD is anticonvulsant; however, its mechanism of action is unclear and likely involves numerous epilepsy drug targets (Devinsky et al. 2016; Pisanti et al. 2017). Many clinically effective anticonvulsants inhibit Na_V_ channels, and purified CBD non-selectively inhibits these channels (Ghovanloo et al. 2018). However, as cannabis contains 500 phytochemicals including phytocannabinoids and terpenes, many cannabis-based products being used to treat epilepsy contain constituents beyond CBD (Nuutinen 2018). Our study shows that a CBD-dominant nutraceutical product that contains a mixture of phytocannabinoids and terpenes potently modulated various functional properties of the sodium channel subtypes Na_V_1.1–Na_V_1.8.

The IC_50_ values of the hemp-derived NP across Na_V_1.1-Na_V_1.7 ranged from 1.6 to 4.2 μg NP/mL, which equates to CBD IC_50_ values of 93–245 nM. Our data show that purified CBD inhibited a subset of Na_V_ channels with IC_50_ values in the micromolar range. Thus, NP is an order of magnitude more potent than purified CBD at these Na_V_ channels, implying that either another component of the NP potently inhibits the channels or that the various components within the NP work cooperatively to inhibit channel function. Whether the other phytocannabinoid or terpene constituents of NP alone directly affect Na_V_ channel subtypes is unknown, although there is evidence that Δ^9^-THC inhibits sodium channels at micromolar concentrations (Turkanis et al. 1991) and linalool inhibits Na_V_ channels at high micromolar concentrations (Leal-Cardoso et al. 2010). Mounting evidence supports synergistic interactions between the many molecular constituents of cannabis-derived products, consistent with the notion of an “entourage effect” (Russo 2019; Russo 2011). Future studies could explore whether an “entourage effect” or synergism between components within the NP contributes to its inhibition of Na_V_ channel function.

NP (3 μg NP/mL) inhibited peak current amplitude across this superfamily of human sodium channels, with the exception Na_V_1.8, where no change was seen, and Na_V_1.3 where peak current amplitude was enhanced. However, hemp seed oil vehicle (3 μg hemp seed oil/mL) also enhanced peak current amplitude of Na_V_1.3 suggesting the effect was not specific to the NP. While postnatal expression of Na_V_1.3 is very low in rodent brain tissue, human tissue distribution studies have shown that Na_V_1.3 is retained in the adult brain (Cheah et al. 2013; Gazina et al. 2010). In adult brain, Na_V_1.3 expression is present in both excitatory neurons and inhibitory interneurons (Whitaker et al. 2001). Further, de novo *SCN3A* that are both GOF and LOF have been implicated in childhood epilepsies (Chen et al. 2015; Holland et al. 2008; Lamar et al. 2017; Vanoye et al. 2014). NP and/or hemp seed oil could provide a potential therapeutic benefit in patients with LOF Na_V_1.3 mutations. NP had no effect on peak current amplitude of Na_V_1.8, which is in contrast to previous work that shows both tonic and use-dependent inhibition of Na_V_1.8 channels in murine primary sensory neurons by 2 μM CBD (Zhang and Bean 2021). Since the highest concentration of CBD examined in the NP was only 175 nM, it may explain why no effect was observed. Future studies could also determine whether interactions between NP and sodium channels exhibit state-dependence.

NP had variable effects on the biophysical properties of activation, steady-state fast inactivation and recovery from fast inactivation for the channel isoforms. NP did not affect the activation properties of Na_V_1.1, Na_V_1.2, Na_V_1.4 or Na_V_1.7; however, it did cause hyperpolarizing shifts in the midpoint of activation for Na_V_1.3 and Na_V_1.8, indicative of enhanced channel availability. A depolarizing shift in the conductance curve for Na_V_1.5 was observed with NP, thus impeding channel function. The overall inhibitory effects of NP on Na_V_1.5, the cardiac sodium channel, provides some cause for concern. However, purified CBD is well tolerated in humans with no clinically significant ECG changes even at peak plasma concentrations >2 μM, consistent with purified CBD only modestly inhibiting Na_V_1.5 (IC_50_ of 3.8 μM) (Ghovanloo et al. 2018). While there is scant data available evaluating plasma exposure levels in patients using nutraceutical hemp extracts, a recent clinical trial reported that a CBD-dominant cannabis herbal extract attained a plasma concentration of ~200 nM in childhood epilepsy patients when dosed up to 10–12 mg/kg CBD per day (Huntsman et al. 2019). No serious adverse events were reported in this study; however, it would be prudent for future safety studies administering CBD-dominant cannabis based-products to incorporate ECG measures. However, it is unlikely that this product would affect sodium channels when administered as a nutraceutical product where a common dosage is 15 mg CBD twice daily, amounting to a ~0.5 mg/kg per day CBD dose (Capano et al. 2020). NP treatment resulted in hyperpolarizing shifts in the midpoint of inactivation for all channel subtypes, except Na_V_1.2. Hyperpolarizing shifts of channel inactivation mean that the channels move into an inactivated state more readily, indicative of the channels being functionally inhibited.

Recovery from steady-state fast inactivation, for Na_V_1.1, Na_V_1.2, Na_V_1.4, Na_V_1.5, Na_V_1.6 and Na_V_1.7, was significantly slower in the presence of NP and is also consistent with functional inhibition of these channels. Notably, these effects appear to be independent of the NP as the hemp seed oil vehicle produced the same pattern of results on channel inactivation, suggesting other components of the hemp seed oil, which is devoid of phytocannabinoids and terpenes, exert these effects.

Our results suggest that NP might be further explored to treat epilepsies which are sensitive to inhibition of voltage-gated sodium channels, including LGS where GOF mutations have been identified in *SCN2A* (Na_V_1.2) and *SCN8A* (Na_V_1.6) (Epi4K. 2013). However, it is difficult to reconcile our observation that NP inhibits Na_V_1.1 channel function with research suggesting that CBD-dominant hemp extracts have anticonvulsant effects in DS patients who in 80% of cases have a LOF *SCN1A* mutation (Na_V_1.1) (Depienne et al. 2009). Use of sodium channel inhibitors is contraindicated in DS because inhibiting Na_V_1.1 would exacerbate LOF *SCN1A* mutations by reducing the neuronal inhibition exerted by GABAergic interneurons (Ogiwara et al. 2007; Stein et al. 2019). Interestingly, in DS patient-derived pluripotent stem cells, 50 nM CBD attenuated the excitability of excitatory neurons and potentiated the excitability of inhibitory neurons, opposing effects resulting in reestablishment of normal network activity (Sun and Dolmetsch 2018). Furthermore, therapies that potentiate Na_V_1.1 channel function abolish seizures and reduce mortality in a mouse model of DS (Richards et al. 2018). It remains possible that the promiscuous target activity of the cannabinoids and terpenes in NP affects other epilepsy-relevant drug targets such as GABA_A_ receptors (Anderson et al. 2019a; Bakas et al. 2017), G-protein coupled receptor 55 (GPR55) (Kaplan et al. 2017) and TRPV1 channels (Gray et al. 2020) which overshadow any deleterious effects of Na_V_1.1 channel inhibition. Alternatively, the brain concentrations of the NP might be insufficient to inhibit Nav1.1 in vivo.

## Conclusion

Our present findings show that a CBD-dominant hemp-derived product potently inhibited Na_V_ channels. NP appeared to more potently inhibit Na_V_ channels based on its CBD content than purified CBD, although this could engender both increased therapeutic efficacy and side-effects in epilepsy patients. Future functional characterization of the components of NP, including phytocannabinoids and terpenes, may reveal a potent constituent or interactions between components to modulate sodium channels.

## Supplementary Information

**Additional file 1.** Ananda Hemp 600 certificate of analysis.

**Additional file 2.** Effects of hemp seed oil on voltage-dependent sodium channel currents.

**Additional file 3.** Effect of hemp seed oil on activation and steady-state fast inactivation (SSFI).

**Additional file 4.** NP does not affect the time constant of fast steady-state inactivation.

**Additional file 5.** The effect of hemp seed oil on the time constant of fast inactivation.

**Additional file 6.** Effect of hemp seed oil on recovery from fast inactivation.

**Additional file 7.** Concentration-response curves for hemp seed oil.

## Data Availability

The data used for this study are available from the corresponding author on reasonable request.

## Abbreviations

CBC: Cannabichromene
CBD: Cannabidiol
CBDA: Cannabidiolic acid
CBDV: Cannabidivarin
CBDVA: Cannabidivarinic acid
CBG: Cannabigerol
CBGA: Cannabigerolic acid
CBN: Cannabinol
DS: Dravet syndrome
GOF: Gain-of-function
IC_50_: Half-maximal inhibitory concentration
ipi: Interpulse intervals
LGS: Lennox-Gastaut syndrome
LOF: Loss-of-function
Na_V_: Voltage-gated sodium
NP: Nutraceutical product
SSFI: Steady-state fast inactivation
Δ^9^-THC: Δ^9^-Tetrahydrocannabinol
THCA: Δ^9^-Tetrahydrocannabinolic acid
THCV: Δ^9^-Tetrahydrocannabidvarin
THCVA: Δ^9^-Tetrahydrocannabidvarinic acid
V_0.5_ act: Voltage-dependence of half-activation
V_0.5_ inact: Voltage-dependence of half-inactivation
