# Supplementary figures and images for: A nutraceutical product, extracted from Cannabis sativa, modulates voltage-gated sodium channel function
Source: J Cannabis Res. 2022 Jun 10;4:30. doi: 10.1186/s42238-022-00136-x (PMC9185959; doi:10.1186/s42238-022-00136-x)

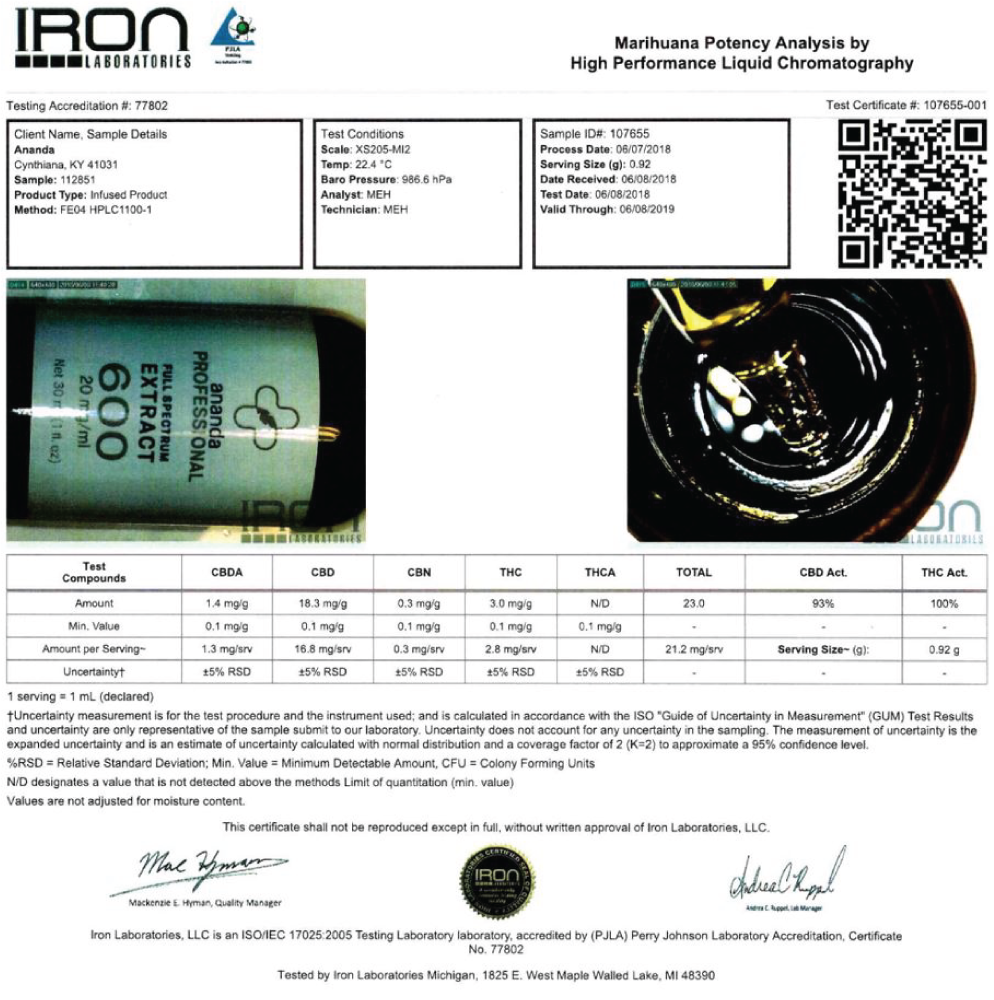

Supplement: Supplementary file 1 — Additional file 1. Ananda Hemp 600 certificate of analysis. [file 42238_2022_136_MOESM1_ESM.tif]

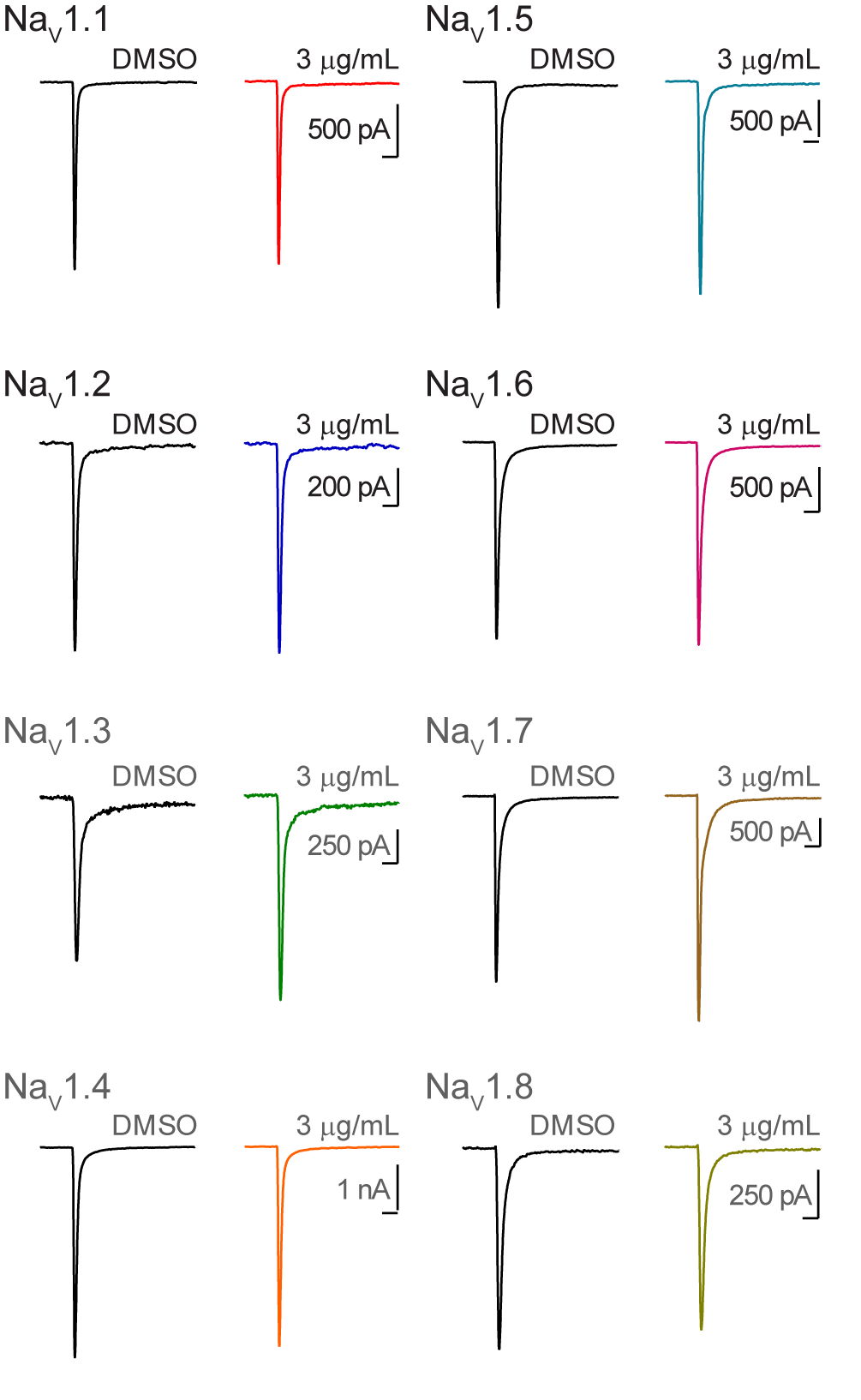

Supplement: Supplementary file 2 — Additional file 2. Effects of hemp seed oil on voltage-dependent sodium channel currents. [file 42238_2022_136_MOESM2_ESM.tif]

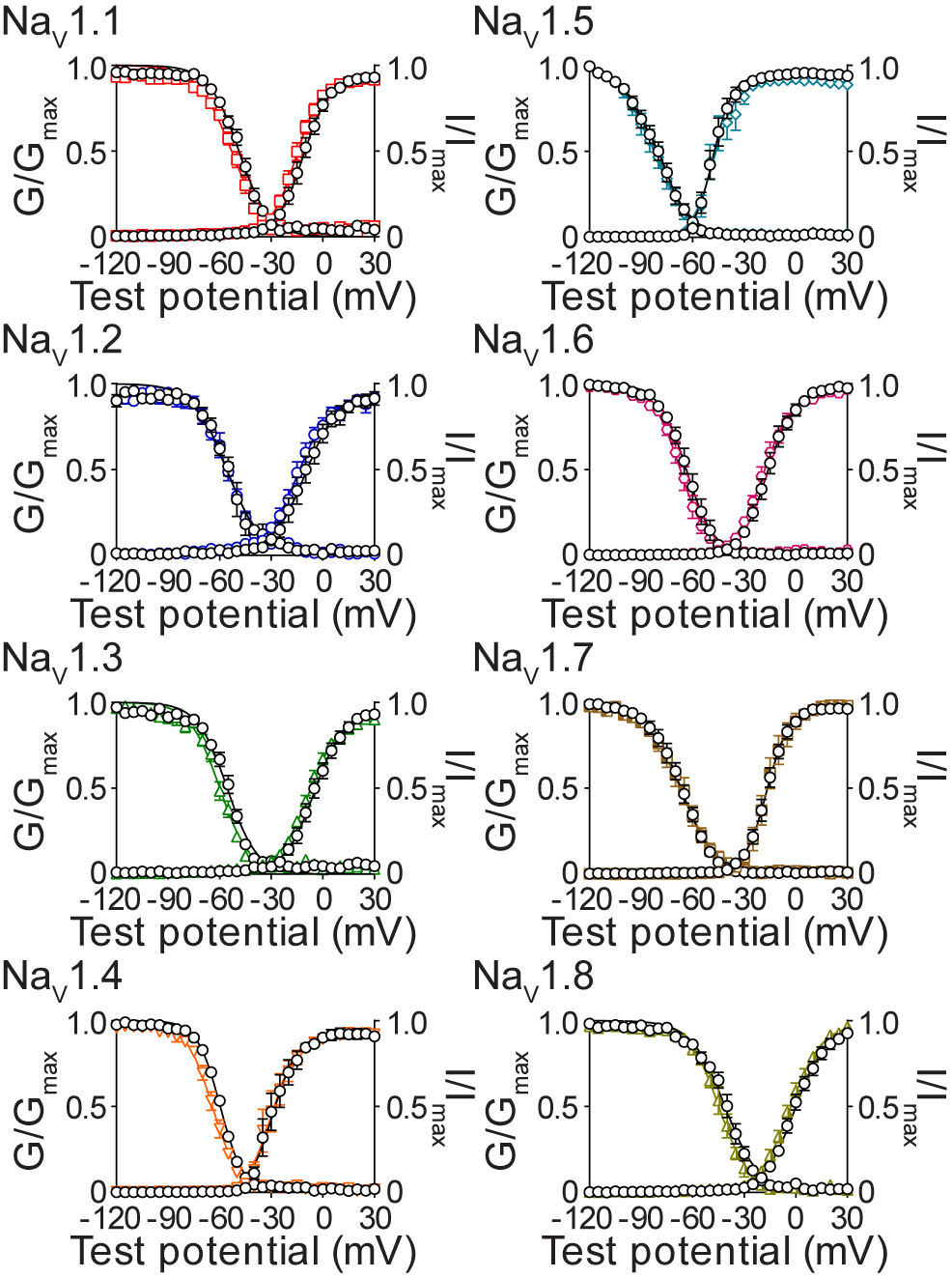

Supplement: Supplementary file 3 — Additional file 3. Effect of hemp seed oil on activation and steady-state fast inactivation (SSFI). [file 42238_2022_136_MOESM3_ESM.tif]

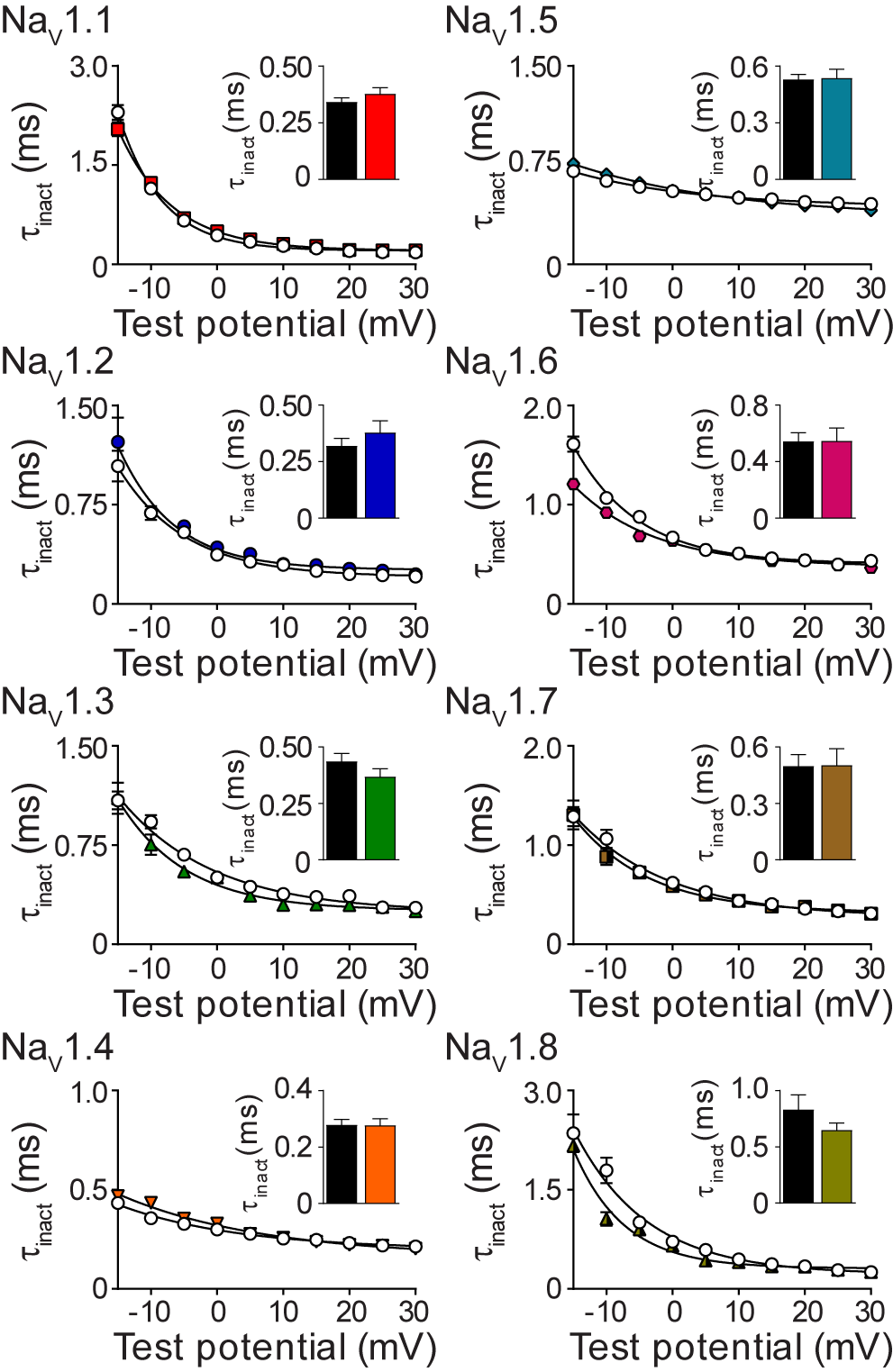

Supplement: Supplementary file 4 — Additional file 4. NP does not affect the time constant of fast steady-state inactivation. [file 42238_2022_136_MOESM4_ESM.tif]

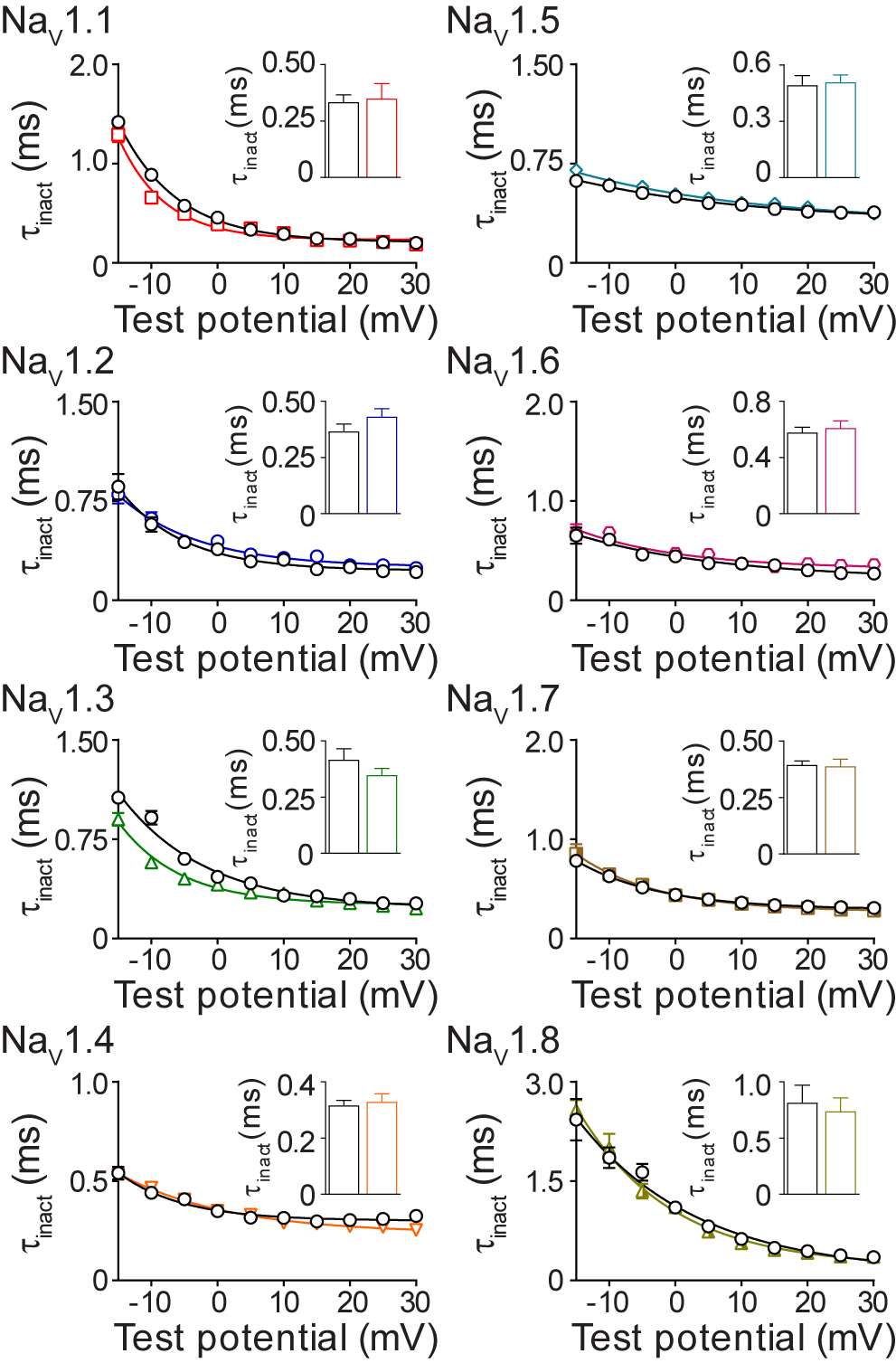

Supplement: Supplementary file 5 — Additional file 5. The effect of hemp seed oil on the time constant of fast inactivation. [file 42238_2022_136_MOESM5_ESM.tif]

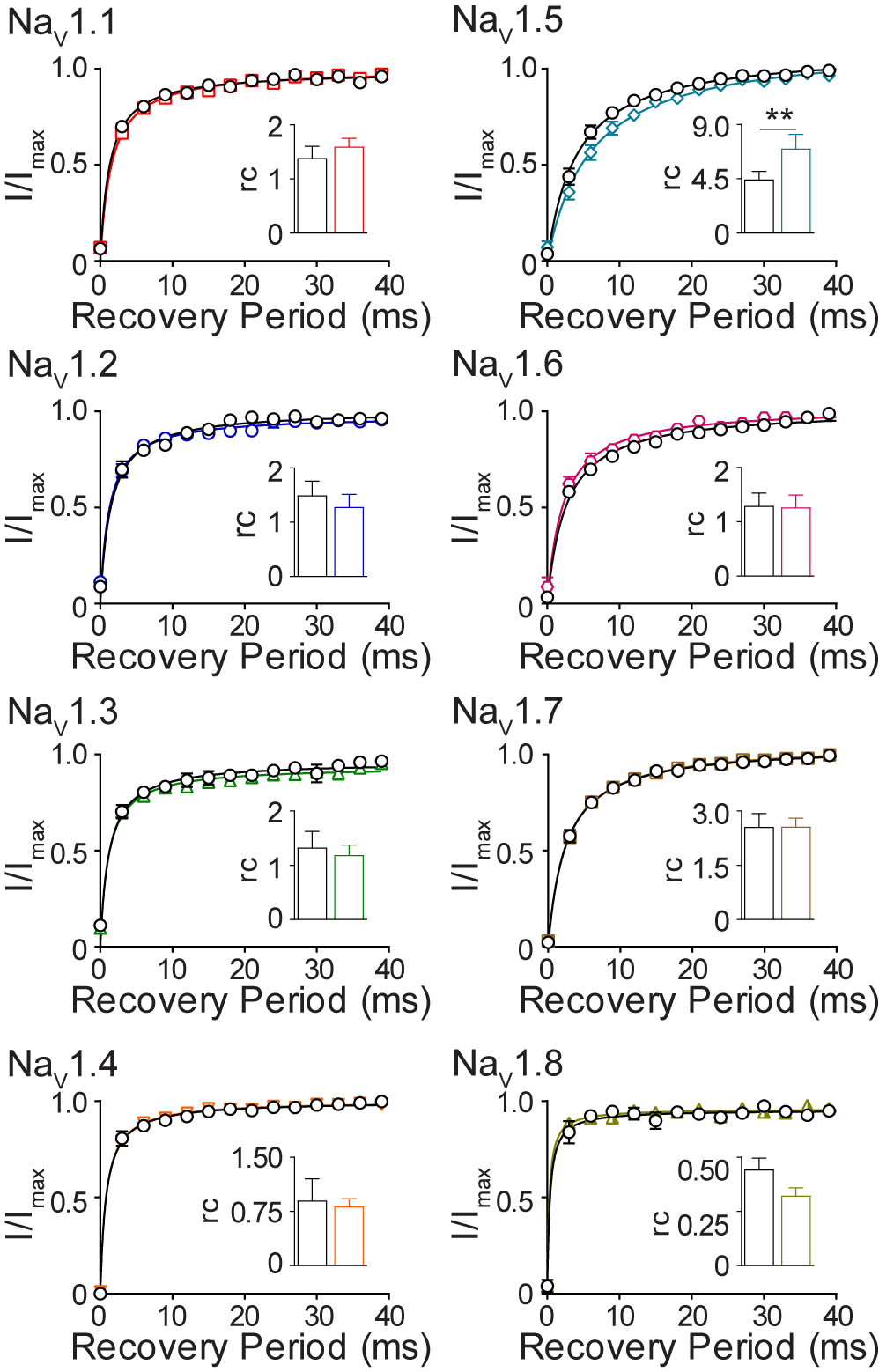

Supplement: Supplementary file 6 — Additional file 6. Effect of hemp seed oil on recovery from fast inactivation. [file 42238_2022_136_MOESM6_ESM.tif]

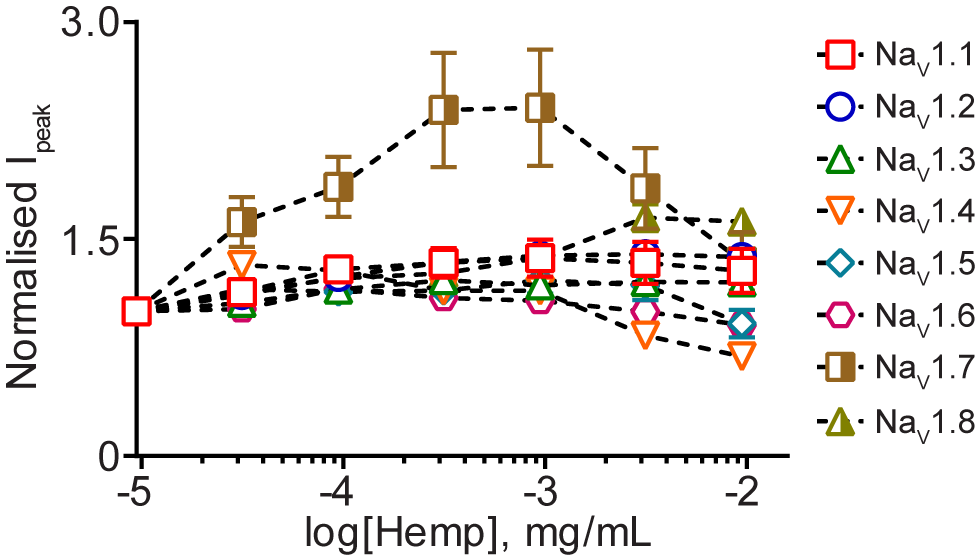

Supplement: Supplementary file 7 — Additional file 7. Concentration-response curves for hemp seed oil. [file 42238_2022_136_MOESM7_ESM.tif]
